# Analysis of the complete chloroplast genomes of *Scutellaria tsinyunensis* and *Scutellaria tuberifera* (Lamiaceae)

**DOI:** 10.1080/23802359.2021.1920491

**Published:** 2021-08-18

**Authors:** Yuanyu Shan, Xiaoying Pei, Shunyuan Yong, Jingling Li, Qiulin Qin, Siyuan Zeng, Jie Yu

**Affiliations:** a College of Horticulture and Landscape Architecture, Southwest University, Chongqing, PR China; b Ministry of Education, Key Laboratory of Horticulture Science for Southern Mountainous Regions, Chongqing, PR China

**Keywords:** *Scutellaria*, chloroplast genome, evolution, hypervariable regions, phylogenetic analysis

## Abstract

*Scutellaria* Linn. is a perennial herb with about 300 species. This genus has high medicinal value and many are used in Traditional Chinese Medicine (TCM). In this study, we sequenced and assembled the complete chloroplast genomes of *Scutellaria tsinyunensis* and *S. tuberifera*. Subsequently, we conducted a comprehensive comparative genomics analysis with 12 other published *Scutellaria* species. These genomes all had a conserved quartile structure, and the gene contents, gene sequences and GC contents are highly similar. The study on the genetic characteristics and nucleotide substitution rate of different genes found that the protein-coding genes of chloroplasts have differed greatly. Most genes are under purifying selection, but the *rps*12 gene may have undergone positive selection. Besides, we identified three hypervariable regions as potential markers for *Scutellaria* taxa, which could play an important role in species identification of *Scutellaria*. Phylogenetic analysis showed that the 14 *Scutellaria* taxa were divided into two major clades. Moreover, the variation of IR regions is closely related to the evolutionary history as was reconstructed based on SNPs. In conclusion, we provided two high-quality chloroplast reference genomes of *Scutellaria*, this reliable information and genomic resources are valuable for developing of efficient DNA barcodes as reconstruction of chloroplast evolutionary history of the genus.

## 1. Introduction

*Scutellaria* Linn. is a perennial herb of about 300 species, which belongs to the family Lamiaceae. *Scutellaria* plants are widely distributed throughout the world except for tropical Africa. Several species from *Scutellaria* are used in Traditional Chinese Medicine (TCM) with the functions of clearing away heat and dampness, purging internal heat, and detoxification (Zhao T et al. 2019). For instance, the dried roots of *S. baicalensis*, also known as ‘Huang Qin’, are used for liver and lung complaints and even used for complementary cancer treatments (EghbaliFeriz et al. 2018; Wang CZ et al. 2020). Phytochemical studies have shown that the main active compounds of *Scutellaria* species are a series of flavonoids, include wogonin, wogonoside, baicalin, and baicalein (Wang ZL et al. 2018; Zhao Q et al. 2019). By now, the research on *Scutellaria* taxa is mainly focused on chemical composition, medicine activity and biological technology (Wang ZL et al. 2018; Zhao Q et al. 2019). In particular for *S. baicalensis*, which is favored for excellent effect in disease treatment. However, the resource identification based on molecular phylogenetic studies is relatively scarce.

Chloroplast genome (referred to as cp genome in the following text) plays an important role in plant photosynthesis (Szabò and Spetea 2017) and are widely used in phylogenetic studies and species identification (Santos and Pereira 2018; Wang A et al. 2018). Due to its conservative genome structure and contents, the cp genome has become an ideal model for evolutionary and comparative genomic studies (Shin et al. 2016). Although the cp genome is relative conserved compared to the nuclear genomes, it also contains highly variable regions that were widely used as molecular markers (Liu ML et al. 2018; Liu X et al. 2018; Pang et al. 2019; Thakur et al. 2019). For instance, *mat*K, *rbc*L, and *trn*H-*psb*A were used as the universal DNA barcodes for distinguishing species (de Vere et al. 2015; Guo et al. 2011; Yu et al. 2021). In a recent study, Zhao et al. (2020) reported 8 cp genomes of *Scutellaria* plants, which have greatly enriched the cp genome resources. However, cp genome sequencing is still inadequate in such a moderately large genus, and the comparative genomic analysis of cp genomes is incomplete.

In our study, we have sequenced two cp genomes of *Scutellaria* species, they are *S. tsinyunensis* C.Y. Wu & S. Chow and *S. tuberifera* C. Y. Wu et C. Chen. Among them, *S. tsinyunensis* is an endangered perennial herb endemic to Mt. Jinyun, Chongqing, China (Li and Hedge 1994). Subsequently, we conducted a comprehensive comparative genomics analysis with 12 other published *Scutellaria* taxa. In particular, we focused on the molecular evolution of chloroplast genomes, such as the expansion/contraction of IR regions, the evolution of protein-coding genes, and the identification of hypervariable regions. The entire cp genome sequences were used as a super-barcode to determine the phylogenetic position of *Scutellaria* plants.

## 2. Materials and methods

### 2.1. Sampling, DNA extraction and sequencing

The fresh leaves of two *Scutellaria* species, *S. tsinyunensis* and *S. tuberifera* were collected from Mt. Jinyun, Chongqing (Geospatial coordinates: N29.842889, E106.394527) and Greenhouse 9, Southwest University, Chongqing (Geospatial coordinates: N29.817767, E106.421054), respectively. The samples have been deposited in the herbarium of Southwest University, Chongqing, China with the accession number: 20200320CQ-1 and 20200320CQ-2, respectively. The total genomic DNA was extracted by using CTAB method (Arseneau et al. 2017). The DNA library with an insert size of 350 bp was constructed using the NEBNext^®^ library building kit (Emerman et al. 2017) and sequenced by using the Hiseq Xten PE150 sequencing platform. Sequencing produced a total of 4.19 G and 5.23 G raw data. A total of 19,816,746 and 22,736,325 raw reads (2 × 150 bp) were obtained. Clean data were obtained by removing low-quality sequences: sequences with a quality value of Q < 19 accounted for more than 50% of the total base, and sequences with more than 5% bases being ‘N’.

### 2.2. Genome assembly and annotation

Genome assembly from the clean data was accomplished utilizing NOVOPlasty version 2.7.2 (Dierckxsens et al. 2017), with a k-mer length of 39 bp and a sequence fragment of the *rbc*L gene from maize as the seed sequence. The average base-coverage was 499.3 (*S. tsinyunensis*) and 506.6 (*S. tuberifera*). Then, we use Geneious version 8.1 (Auckland, New Zealand) (Kearse et al. 2012) to map all clean reads to the assembled genome sequence to verify whether the spliced contigs were correct. The cp genome was annotated initially by using CPGAVAS2 (Shi et al. 2019) using the reference dataset of 2544-plastomes. Geseq was then used to confirm the annotation results (Tillich et al. 2017). Furthermore, the annotations with problems were manually edited by using Apollo (Misra and Harris 2005).

### 2.3. Sequence analysis and genome comparison

The GC content was conducted by using the cusp program provided by EMBOSS version 6.3.1 (Rice et al. 2000). IRscope (https://irscope.shinyapps.io/irapp/) was used for visualizing the IR boundaries in these cp genomes (Amiryousefi et al. 2018). A total of 78 orthologous genes and 89 intergenic spacer regions (IGSs) among 14 *Scutellaria* species were identified and extracted by using Phylosuite version 1.2.1 (Zhang et al. 2020). The corresponding nucleotide sequences were aligned by using MAFFT version 7.450 (https://mafft.cbrc.jp/alignment/server/) (Rozewicki et al. 2019) implemented in Phylosuite. We used MEGA version 6.0 (Tamura et al. 2013) to calculate the percentage of variable sites (PV) in protein-coding genes and the pairwise K2-P distance in IGSs. Then, we used DnaSP version 6.0 (Rozas et al. 2017) to calculate the nucleotide diversity (Pi) among the protein-coding sequence.

### 2.4. Nucleotide substitution rate analysis

The protein-coding sequences in the previous step were processed in parallel. We used the CODEML module in PAML version 4.9 (Yang 2007) to estimate rates of nucleotide substitution, including dN (nonsynonymous), dS (synonymous), and the ratio of nonsynonymous to synonymous rates (dN/dS). The detailed parameters were: CodonFreq = 2 (F3 × 4 model); model = 0 (allowing a single dN/dS value to vary among branches); cleandata = 1 (remove sites with ambiguity data); other parameters in the CODEML control file were left at default settings. The phylogeny tree structure of each gene was generated by using the maximum-likelihood (ML) method implemented in RaxML version 8.2.4 (Stamatakis 2014).

### 2.5. Phylogenetic analysis

The cp genome sequences of 14 species belonging to Lamiaceae were downloaded from GenBank (Table S1). Two species (*Lamium album* and *Stachys byzantina*) were used as outgroups. A total of 16 complete cp genome sequences were aligned by using MAFFT version 7.450 online version with default setting (Rozewicki et al. 2019). These aligned sequences were used to construct the phylogenetic trees by using the ML method implemented in RaxML version 8.2.4 (Stamatakis 2014). The parameters were ‘raxmlHPC-PTHREADS-SSE3 -f a -N 1000 -m GTRGAMMA -x 551314260 -p 551314260’. The bootstrap analysis was performed with 1000 replicates.

**Table 1.** Basic features of the 14 cp genomes from *Scutellaria*.

Species	Accession Number	Length (bp)				GC contents (%)				Number of genes			
		Total	LSC	SSC	IR	Total	LSC	SSC	IR	Total	Protein	*tRNA*	*rRNA*
*S. baicalensis*	MF521632.1	151,824	83,976	17,338	25,255	38.3	36.3	32.7	43.6	134	89	37	8
*S. insignis*	NC_028533.1	151,908	83,913	17,517	25,239	38.4	36.5	32.6	43.6	134	89	37	8
*S. indica var. coccinea*	MN047312.1	151,956	83,951	17,537	25,234	38.3	36.4	32.5	43.6	134	89	37	8
*S. kingiana*	MN128389.1	152,395	84,608	17,305	25,241	38.3	36.3	32.4	43.6	132	87	37	8
*S. altaica*	MN128387.1	151,779	83,984	17,327	25,234	38.3	36.3	32.6	43.6	134	89	37	8
*S. amoena var. amoena*	MN128386.1	151,833	84,001	17,340	25,246	38.3	36.3	32.7	43.6	134	89	37	8
*S. calcarata*	MN128385.1	152,033	84,023	17,532	25,239	38.4	36.4	32.6	43.6	134	89	37	8
*S. mollifolia*	MN128384.1	152,417	84,432	17,569	25,208	38.3	36.4	32.6	43.6	134	89	37	8
*S. orthocalyx*	MN128383.1	152,071	84,072	17,519	25,240	38.4	36.4	32.6	43.6	134	89	37	8
*S. przewalskii*	MN128382.1	151,675	83,891	17,320	25,232	38.3	36.4	32.6	43.6	134	89	37	8
*S. quadrilobulata*	MN128381.1	152,066	84,052	17,544	25,235	38.3	36.4	32.5	43.6	134	89	37	8
*S. lateriflora*	NC_034693.1	152,283	84,340	17,465	25,239	38.3	36.3	32.5	43.6	134	89	37	8
*S. tsinyunensis*	MT544405.1	152,089	84,110	17,533	25,223	38.4	36.4	32.6	43.6	134	89	37	8
*S. tuberifera*	MW376477.1	152,332	84,268	17,570	25,247	38.3	36.3	32.5	43.6	134	89	37	8

## 3. Results

### 3.1. General features of cp genomes

The cp genomes of *Scutellaria* species are characterized by a typical circular DNA molecule with the length of 151,675–152,417 bp. It has a conservative quartile structure which is composed of a LSC region (83,891–84,608 bp), an SSC region (17,305–17,570 bp), and a pair of IR regions (25,208–25,255 bp) (Table 1). The GC content analysis showed that the overall GC contents ranged from 38.3% to 38.4% in the 14 cp genomes.

**Figure 1.**
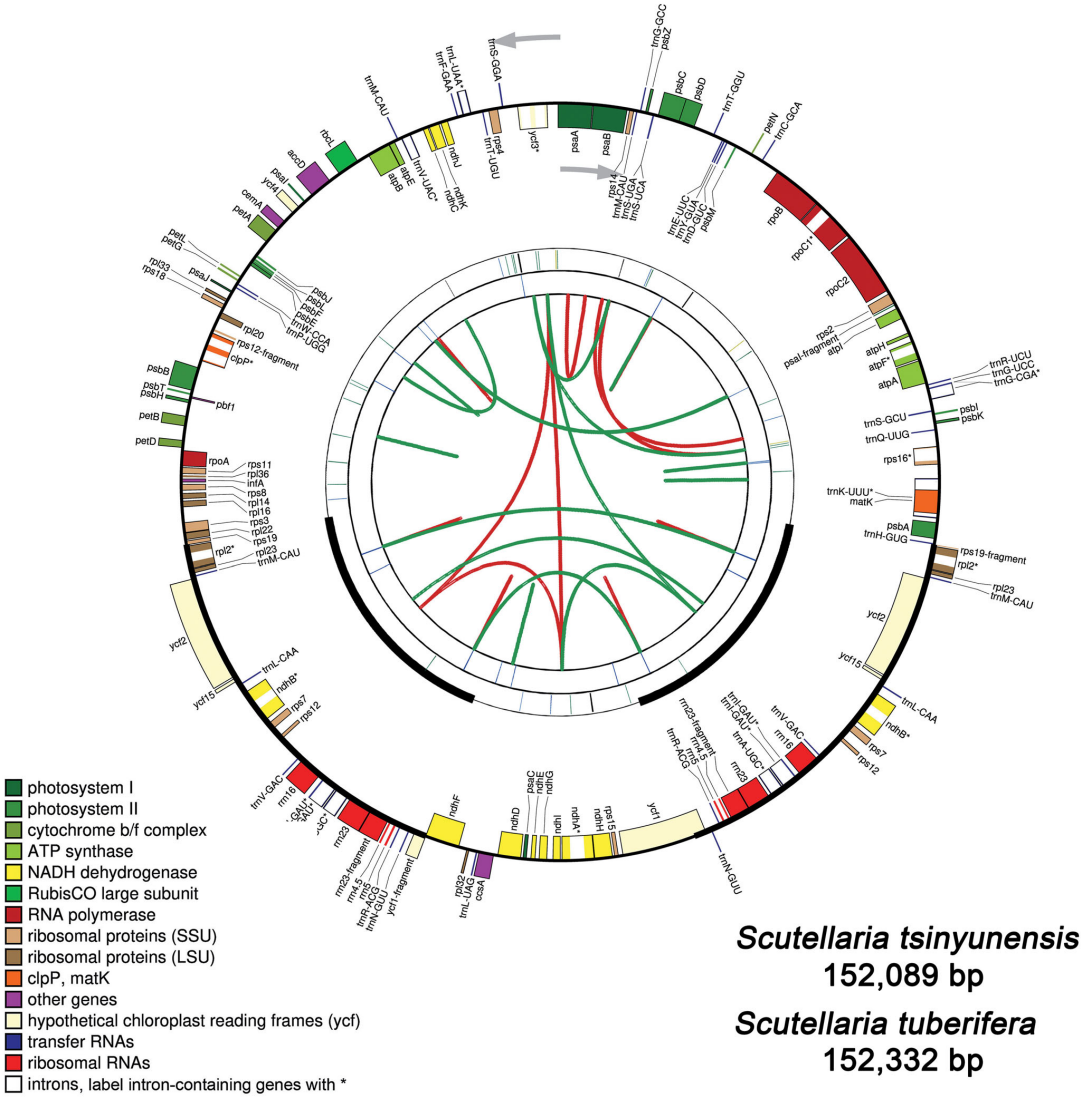
Graphic representation of features identified in the cp genomes of *Scutellaria* plants by using CPGAVAS2. Taking *S. tsinyunensis* as an example, the map contains four rings. From the center going outward, the first circle shows the forward and reverse repeats connected with red and green arcs, respectively. The next circle shows the tandem repeats marked with short bars. The third circle shows the microsatellite sequences identified using MISA. The fourth circle is drawn using drawgenemap and shows the gene structure on the cp genomes. The genes were colored based on their functional categories, which are shown in the left corner. Label intron-containing genes with *.

The cp genomes encode a large number of genes. Take *S. tsinyunensis* for example, the cp genomes comprise 134 genes. Among which, 114 are unique genes, including 80 protein-coding genes, four *rRNAs*, and 30 *tRNAs* (Table 2). Figure 1 shows the schematic diagram of the cp genomes of *S. tsinyunensis*. This result is similar to that of other species in this genus (Jiang et al. 2017; Lee and Kim 2019). In one particular case, two protein-coding genes of *S. kingiana*, *ndhD*, and *ndhF*, are encounter termination codons in advance within the coding frame. As two pseudogenes, they cannot translate the normal protein products. The two genes were not included in subsequent analysis.

**Table 2.** Gene contents of the cp genomes in *Scutellaria* plants.

Category of genes	Group of genes	Name of genes
	*rRNA*	*rrn*16S (x2), *rrn*23S (x2), *rrn*5S (x2), *rrn4.*5S (x2)
	*tRNA*	30 unique tRNA genes (6 contain an intron)
Photosynthesis	Subunits of ATP synthase	*atp*A, *atp*B, *atp*E, *atp*F, *atp*H, *atp*I
	Subunits of photosystem II	*psb*A, *psb*B, *psb*C, *psb*D, *psb*E, *psb*F, *psb*H, *psb*I, *psb*J, *psb*K, *psb*L, *psb*M, *psb*N, *psb*T, *psb*Z
	Subunits of NADH-dehydrogenase	*ndh*A, *ndh*B (x2), *ndh*C, *ndh*D, *ndh*E, *ndh*F, *ndh*G, *ndh*H, *ndh*I, *ndh*J, *ndh*K
	Subunits of cytochrome b/f complex	*pet*A, *pet*B, *pet*D, *pet*G, *pet*L, *pet*N
	Subunits of photosystem I	*psa*A, *psa*B, *psa*C, *psa*I, *psa*J
	Subunit of rubisco	*rbc*L
Self-replication	Large subunit of ribosome	*rpl*14, *rpl*16, *rpl*2 (x2), *rpl*20, *rpl*22, *rpl*23 (x2), *rpl*32, *rpl*33, *rpl*36
	DNA dependent RNA polymerase	*rpo*A, *rpo*B, *rpo*C1, *rpo*C2
	Small subunit of ribosome	*rps*11, *rps*12 (x2), *rps*14, *rps*15, *rps*16, *rps*18, *rps*19, *rps*2, *rps*3, *rps*4, *rps*7(x2), *rps*8
Other genes	Subunit of Acetyl-CoA-carboxylase	*acc*D
	c-type cytochrome synthesis gene	*ccs*A
	Envelop membrane protein	*cem*A
	Protease	*clp*P
	Translational initiation factor	*inf*A
	Maturase	*mat*K
Unknown	Conserves open reading frames	*ycf*1, *ycf*15 (x2), *ycf*3, *ycf*2 (x2), *ycf*4
	Gene Fragments (pseudogene)	*ycf*1, *rps*19, *ndh*D*, *ndh*F*

*Note*. The ‘(x2)’ indicates that the gene located in the IRs and thus had two complete copies. The ‘*’ indicates that it was a pseudogene only in S. *kingiana*.

### 3.2. Contraction and expansion analysis of IR regions

**Figure 2.**
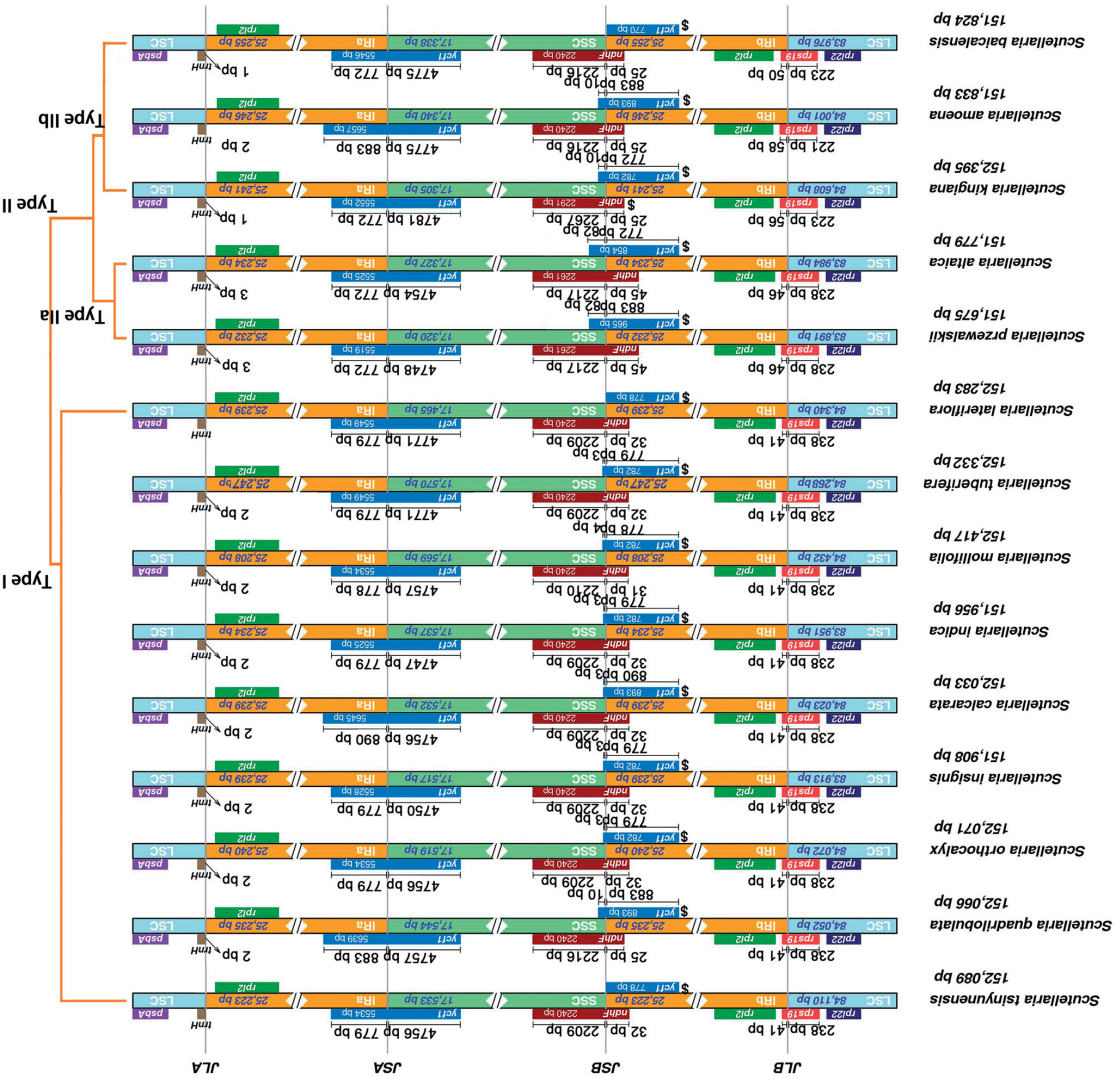
Comparison of the borders among LSC, SSC, and IR regions of 14 analyzed species. The genes around the borders are shown above or below the main line. The JLB, JSB, JSA, and JLA represent junction sites of LSC/IRb, IRb/SSC, SSC/IRa, and IRa/LSC, respectively.

We observed four genes are span the boundary regions in all 14 species, they are *trn*H, *rps*19, *ndh*F, and *ycf*1 (Figure 2). Extensively comparative analysis observed the location of these four genes of *Scutellaria* species is slightly different. Based on these differences, we divide them into two types (three subtypes). For gene *rps*19, it overlaps with the IRb regions by 41 bp in type I. However, in type II, the overlap is 46 bp (type IIa) or more than 50 bp (type IIb). For gene *ndh*F, most sequences are located in SSC regions, it also overlaps with the IRb regions by 32 bp in type I except for *S. quadrilobulata* (25 bp). In type II, the overlap is 45 bp (type IIa) or 25 bp (type IIb). The variation of *ycf*1 genes is quite different, and it did not show an obvious classified pattern. It may be related to the high mutation rates of *ycf*1. It is worth noting that *ndh*F gene is a pseudogene in *S. kinglana*.

Interestingly, the *ndh*F genes cross the border of IRb/SSC, and we observed overlaps of *ndh*F and the first copy of *ycf*1. The length of the overlapping regions ranged from 25 to 35 bp in type I and type IIb, but over 120 bp in two species from type IIa, indicating that type I is close to type IIb, and they are quite different from type IIa.

### 3.3. Genetic characteristics of protein-coding genes

**Figure 3.**
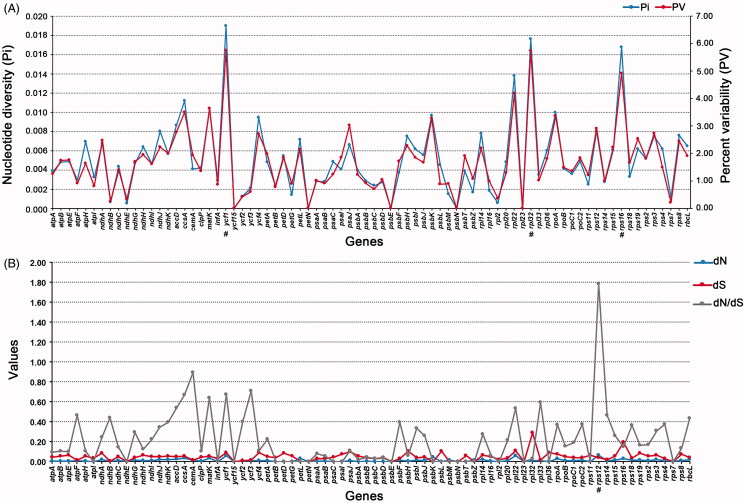
Genetic characteristics among protein-coding genes of chloroplast in *Scutellaria*. A. Nucleotide diversity (Pi) and percent variability (PV) of 78 orthologous genes. B. Estimations of nonsynonymous (dN), synonymous (dS) substitution rates, and their ratio (dN/dS) of 78 orthologous genes. A few maximum values were marked with ‘#’.

In our study, the Pi value and PV value were highly similar in all 78 genes (Figure 3(A)). The Pi value (0.0190) and PV value (5.7550) of *ycf*1 were all the highest. Other genes with high nucleotide polymorphism were *rpl*32 (0.0176, 5.7471), *rps1*6 (0.0168, 4.9242), and *rpl*22 (0.0138, 4.1850). The Pi value and PV value were given in parentheses one by one, respectively (Table S2). Five genes (*ycf*15, *pet*N, *psb*E, *psb*N, and *rpl*23) did not have any variable sites and they are highly conserved.

The rates of synonymy (dS) and non-synonymous (dN) substitution rates and their ratios (dN/dS) of 78 orthologous genes were estimated to detect the heterogeneity of substitution rates. Among the 78 genes, *rps*12, *ycf*1, *rpl*22, and *psb*K had higher dN values, which were 0.0652, 0.0604, 0.0591, and 0.0432, respectively. The dS value of *rpl*32 was the highest at 0.2896 (Figure 3(B), Table S3). The dN/dS value of most genes was less than 0.6, indicating that they have been under purifying selection during evolution. It is worth noting that the dN/dS value of *rps*12 gene reaches 1.7814, which is likely to undergo positive selection. Other genes with higher dN/dS values are *cem*A (0.8926), *ycf*3 (0.7090), *ycf*1 (0.6734), *ccs*A (0.6698), and *mat*K (0.6406), which are all active genes in the process of evolution.

**Table 3.** Mean K2-P **d**istance of 89 IGS of cp genomes from *Scutellaria*.

Number	IGS	Mean K2-P distance	Number	IGS	Mean K2-P distance
1	*acc*D-*psa*I	1.0297	46	*rpl*22-*rps*19	0.0000
2	*atp*A-*atp*F	0.6358	47	*rpl*23-*rpl*2	0.0000
3	*atp*B-*rbc*L	0.4010	48	*rpl*2-*rpl*23	0.0000
4	*atp*F-*atp*H	1.2234	49	*rpl*32-*trn*L-UAG	10.5154
5	*atp*H-*atp*I	1.4355	50	*rpl*33-*rps*18	1.3296
6	*atp*I-*rps*2	1.3093	51	*rpo*A-*rps*11	1.4957
7	*cem*A-*pet*A	0.6834	52	*rpo*B-*trn*C-GCA	1.5659
8	*clp*P-*psb*B	1.3886	53	*rpo*C1-*rpo*B	0.0000
9	*inf*A-*rps*8	1.4089	54	*rpo*C2-*rpo*C1	1.3877
10	*mat*K-*rps*16	2.2641	55	*rps*14-*psa*B	0.0000
11	*ndh*A-*ndh*H	0.0000	56	*rps*15-*ycf*1	2.4660
12	*ndh*B-*rps*7	0.0529	57	*rps*18-*rpl*20	2.0607
13	*ndh*B-*trn*L-CAA	0.2923	58	*rps*19-*rpl*2	0.4941
14	*ndh*C-*trn*V-UAC	1.6266	59	*rps*2-*rpo*C2	1.1111
15	*ndh*E-*ndh*G	2.0309	60	*rps*3-*rpl*22	0.0000
16	*ndh*F-*rpl*32	5.8178	61	*rps*4-*trn*T-UGU	1.8048
17	*ndh*G-*ndh*I	2.4841	62	*rps*7-*ndh*B	0.0529
18	*ndh*H-*rps*15	0.9164	63	*rps*7-*trn*V-GAC	0.1535
19	*ndh*I-*ndh*A	0.0000	64	*rps*8-*rpl*14	1.6979
20	*ndh*J-*ndh*K	0.3573	65	*trn*A-UGC-*trn*I-GAU	0.8327
21	*pet*B-*pet*D	1.1184	66	*trn*C-GCA-*pet*N	3.0135
22	*pet*D-*rpo*A	1.1514	67	*trn*D-GUC-*trn*Y-GUA	2.1403
23	*pet*G-*trn*W-CCA	0.4677	68	*trn*F-GAA-*ndh*J	1.3359
24	*pet*L-*pet*G	0.3304	69	*trn*H-GUG-*psb*A	3.8331
25	*pet*N-*psb*M	1.6852	70	*trn*I-CAU-*rpl*23	0.0900
26	*psa*A-*ycf*3	1.4501	71	*trn*I-GAU-*trn*A-UGC	0.8352
27	*psa*B-*psa*A	0.0000	72	*trn*L-CAA-*ndh*B	0.2923
28	*psa*C-*ndh*E	2.9548	73	*trn*L-CAA-*ycf*15	0.3907
29	*psa*I-*ycf*4	1.1392	74	*trn*L-UAA-*trn*F-GAA	1.7191
30	*psa*J-*rpl*33	1.5216	75	*trn*L-UAG-*ccs*A	6.1056
31	*psb*A-*trn*K-UUU	1.1092	76	*trn*P-UGG-*psa*J	0.3429
32	*psb*B-*psb*T	1.6846	77	*trn*R-ACG-*trn*N-GUU	0.5666
33	*psb*E-*pet*L	1.4515	78	*trn*R-UCU-*atp*A	0.8224
34	*psb*F-*psb*E	0.0000	79	*trn*S-GGA-*rps*4	1.3310
35	*psb*H-*pet*B	0.6431	80	*trn*T-UGU-*trn*L-UAA	1.9092
36	*psb*I-*trn*S-GCU	2.3382	81	*trn*V-GAC-*rps*7	0.1444
37	*psb*K-*psb*I	1.4205	82	*trn*W-CCA-*trn*P-UGG	1.3533
38	*psb*L-*psb*F	0.0000	83	*ycf*15-*trn*L-CAA	0.3907
39	*psb*M-*trn*D-GUC	1.0245	84	*ycf*15-*ycf*2	0.1262
40	*psb*N-*psb*H	1.0605	85	*ycf*1-*trn*N-GUU	0.7302
41	*psb*T-*psb*N	1.8007	86	*ycf*2-*trn*I-CAU	0.5687
42	*rbc*L-*acc*D	1.7944	87	*ycf*2-*ycf*15	0.1218
43	*rpl*14-*rpl*16	1.6116	88	*ycf*3-*trn*S-GGA	1.1564
44	*rpl*16-*rps*3	3.3620	89	*ycf*4-*cem*A	1.2918
45	*rpl*20-*clp*P	1.0954	–	–	–

### 3.4. Identification of hypervariable regions

**Figure 4.**
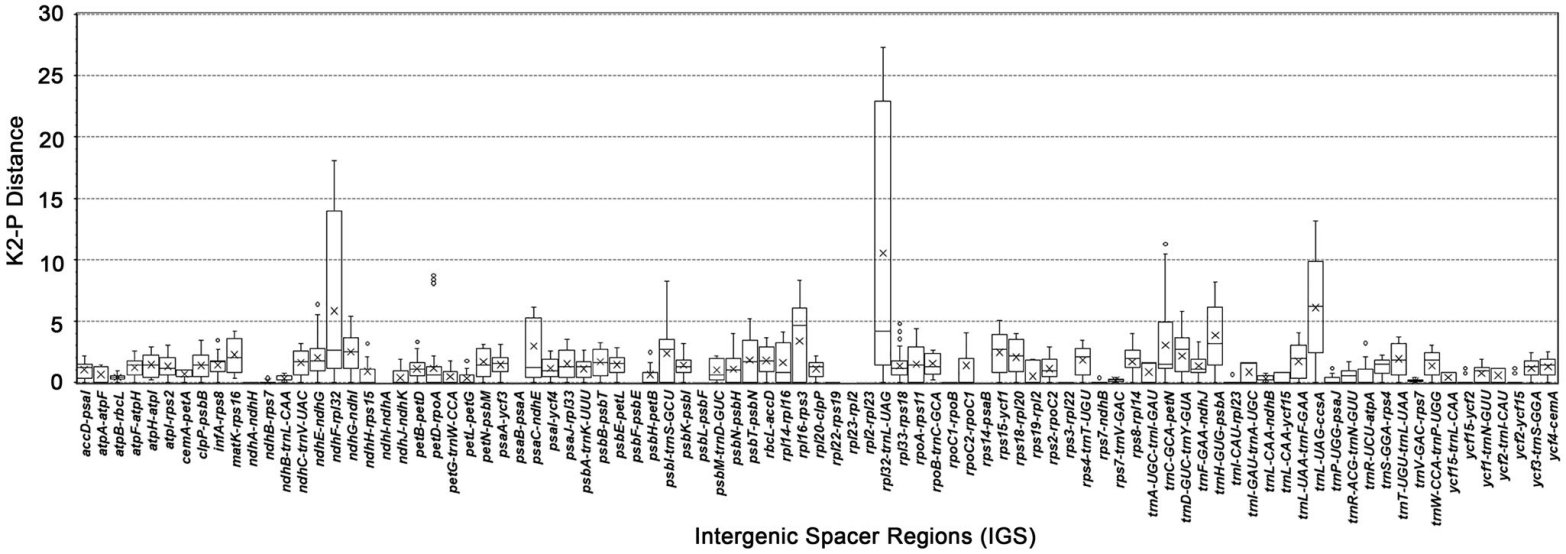
Boxplot for pairwise comparison of the K2-P distance among 89 intergenic spacers (IGS) of 14 *Scutellaria* species. The ‘× in each boxplot represents the average K2-P distance. Three IGS had mean K2-P distance over 5, they are *ndh*F-*rpl*32 (5.8178), *trn*L-UAG-*ccs*A (6.1056) and *rpl*32-*trn*L-UAG (10.5154).

Considering that the protein-coding genes are extremely conserved, we are more focused on the IGSs. As shown in Figure 4, the K2P distances of the 89 IGSs were quite different. The maximum, minimum and mean K2-P distance showed significant differences in three IGS, which are *ndh*F-*rpl*32 (5.8178), *trn*L-UAG-*ccs*A (6.1056), and *rpl*32-*trn*L-UAG (10.5154). The mean was given in the parentheses, and the details are shown in Table 3. The above three IGSs could be used as potential DNA barcodes. Other IGSs with larger differences were *trn*H-GUG-*psb*A, *rpl*16-*rps*3, *trn*C-GCA-*pet*N, and *psa*C-*ndh*E, which could be used as candidate hypervariable regions.

### 3.5. Phylogenetic analysis

**Figure 5.**
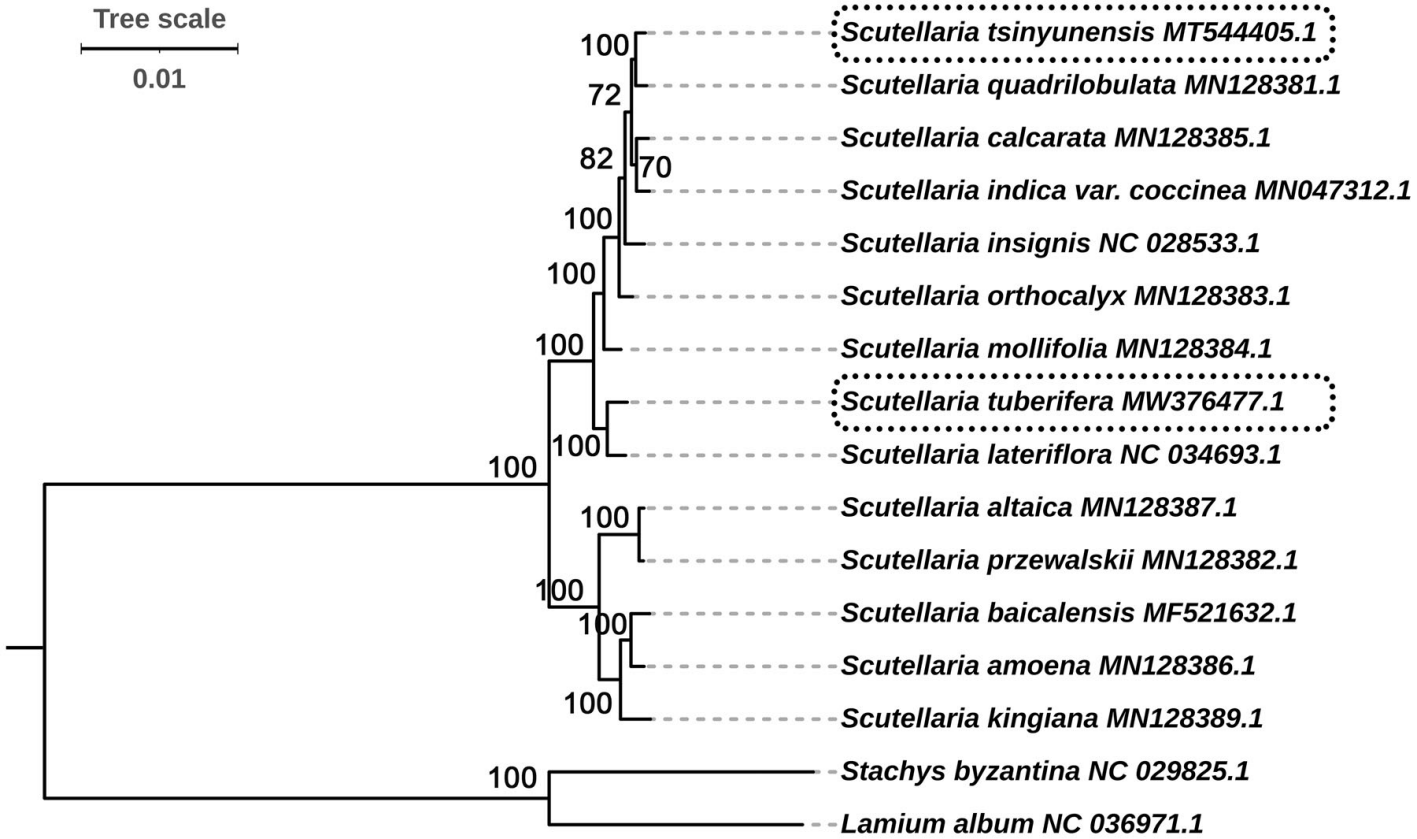
Phylogenetic relationships of *Scutellaria* species inferred using maximum-likelihood (ML) method. The phylogenetic tree was constructed using complete cp genome sequences. The number at the bottom of the scale, 0.01, means that the length of the branch represents the replacement frequency of bases at each site of the genome at 0.01. Bootstrap values were calculated from 1000 replicates. Two taxa, namely, *Lamium album* and *Stachys byzantina* were used as outgroups.

In this study, we selected two outgroups and analyzed the phylogenetic relationships of 14 *Scutellaria* species. The 16 complete cp genome sequences were used for constructing a ML tree. The phylogenetic trees have high bootstrap support values (100) on most nodes except for three nodes, showing the reliability of the phylogeny recovered (Figure 5). Our phylogenetic trees displayed two clades clearly, and then further diversified into different subclades. Among the two clades, five species (*S. baicalensis*, *S. Amoena*, *S. Kingiana*, *S. Altaica*, and *S. Przewalskii*) were clustered, and the other nine *Scutellaria* taxa clustered together. In the two species that we sequenced, *S. tsinyunensis* had the closest relationship with *S. quadrilobulata*, and *S. tuberifera* had the closest relationship with *S. lateriflora*. These results exhibited that the whole cp genome sequences can be used as a super-barcode for species identification with extremely high resolution at the species level.

## 4. Discussion

Here, we sequenced and assembled the complete chloroplast genomes of *S. tsinyunensis and S. tuberifera*, which were highly similar to previously published one (Jiang et al. 2017; Lee and Kim 2019). This result suggested the cp genomes were highly conserved in *Scutellaria*.

The contraction and expansion of IR regions are considered to be an important reason for the length diversity in cp genomes (Goulding et al. 1996). In the comparative analysis, although no significant differences were observed in *Scutellaria*, we were able to divide the cp genomes into several basic types based on the subtle differences of IR regions. It is worth noting that the dynamic changes of genes near the IR boundary are consistent with the topology of the phylogenetic tree, indicating that, structure variation, e.g. the shift of IR boundaries, reflected similar evolutionary history as was reconstructed based on SNPs.

In previous studies, the evolution of plastid protein-coding genes in *Scutellaria* was rarely involved. The 14 samples allowed us to conduct a wide range of plastid gene studies in *Scutellaria*, and we calculated the nucleotide substitution rates to understand the evolution rates of plastid genes in *Scutellaria* species. We found a wide range of heterogeneous evolutionary rates of the plastid genes. Some genes have higher mutation rates, such as *ycf*1, *rps*16, and *rpl*32. While some are highly conserved and have no mutation sites. This difference in the rates of evolution is important for studying the molecular evolution of chloroplasts, because it is usually related to the purifying or positive selection of genes during evolution. In particular, we observed that the dN/dS value of *rps*12 gene was greater than 1, suggesting that the gene might have undergone positive selection during evolution processes. As part of the 30S ribosomal subunit, *rps*12 had the function of rRNA binding, and it is also the only trans-splicing gene in the chloroplast. By contrast, the evolution rate of non-coding regions of the cp genome is generally higher than that of protein-coding regions. The study of non-coding regions or IGSs is helpful for us to find appropriate species-specific DNA barcodes. Based on the results of K2-P distance, we recommended three hypervariable regions, including *ndh*F-*rpl*32, *trn*L-UAG-*ccs*A, and *rpl*32-*trn*L-UAG. These markers could be used to distinguish different species of the genus or even different individuals of the same species.

In summary, our results enrich the data on the cp genomes of *Scutellaria* and provide the basis for the phylogenetic reconstruction of *Scutellaria*. We have carried out in-depth studies on plastid genes and deepened our understanding of plastid genes of *Scutellaria* taxa.

## Supplementary Material

Supplemental Material

## Data Availability

The raw sequencing data and two genome sequences have been deposited in NCBI (https://www.ncbi.nlm.nih.gov/) with accession number: PRJNA680174, MT544405.1 (*S. tsinyunensis*), and MW376477.1 (*S. tuberifera*). The sample has been deposited in the herbarium of Southwest University, Chongqing, China with the accession number: 20200320CQ-1 and 20200320CQ-2, respectively.
