# Supplementary material for: Analysis of the complete chloroplast genomes of Scutellaria tsinyunensis and Scutellaria tuberifera (Lamiaceae)
Source: Mitochondrial DNA B Resour. 2021 Aug 18;6(9):2672–80. doi: 10.1080/23802359.2021.1920491 (PMC8381982; doi:10.1080/23802359.2021.1920491)
Supplement: Supplemental Material [file TMDN_A_1920491_SM4731.docx]

# Supplementary Tables

**Table S1** List of chloroplast genomes used for phylogenetic analysis.

| NO. | Species | Family | Subfamily | Accession Number |
| --- | --- | --- | --- | --- |
| 1 | *Lamium album* | Lamiaceae | Lamioideae | NC_036971.1 |
| 2 | *Stachys byzantina* | Lamiaceae | Lamioideae | NC_029825.1 |
| 3 | *Scutellaria baicalensis* | Lamiaceae | Scutellarioideae | MF521632.1 |
| 4 | *Scutellaria indica var. coccinea* | Lamiaceae | Scutellarioideae | MN047312.1 |
| 5 | *Scutellaria insignis* | Lamiaceae | Scutellarioideae | NC_028533.1 |
| 6 | *Scutellaria lateriflora* | Lamiaceae | Scutellarioideae | NC_034693.1 |
| 7 | *Scutellaria moena var. moena* | Lamiaceae | Scutellarioideae | MN128386.1 |
| 8 | *Scutellaria kingiana* | Lamiaceae | Scutellarioideae | MN128389.1 |
| 9 | *Scutellaria altaica* | Lamiaceae | Scutellarioideae | MN128387.1 |
| 10 | *Scutellaria przewalskii* | Lamiaceae | Scutellarioideae | MN128382.1 |
| 11 | *Scutellaria calcarata* | Lamiaceae | Scutellarioideae | MN128385.1 |
| 12 | *Scutellaria quadrilobulata* | Lamiaceae | Scutellarioideae | MN128381.1 |
| 13 | *Scutellaria orthocalyx* | Lamiaceae | Scutellarioideae | MN128383.1 |
| 14 | *Scutellaria mollifolia* | Lamiaceae | Scutellarioideae | MN128384.1 |
| 15 | *Scutellaria tsinyunensis* | Lamiaceae | Scutellarioideae | MT544405.1 |
| 16 | *Scutellaria tuberifera* | Lamiaceae | Scutellarioideae | MW376477.1 |

**Table S2** Pi and PV values of 78 orthologous genes identified in 14 *Scutellaria* Plants.

| Genes | Variable Sites | Aligned length | PV (%) | Pi | Genes | Variable Sites | Aligned length | PV (%) | Pi |
| --- | --- | --- | --- | --- | --- | --- | --- | --- | --- |
| *atp*A | 19 | 1521 | 1.25 | 0.0038 | *psb*C | 10 | 1419 | 0.7 | 0.0024 |
| *atp*B | 26 | 1494 | 1.74 | 0.0048 | *psb*D | 11 | 1059 | 1.04 | 0.0027 |
| *atp*E | 7 | 399 | 1.75 | 0.0049 | *psb*E | 0 | 249 | 0 | 0 |
| *atp*F | 5 | 552 | 0.91 | 0.0029 | *psb*F | 2 | 117 | 1.71 | 0.0037 |
| *atp*H | 4 | 243 | 1.65 | 0.0070 | *psb*H | 5 | 219 | 2.28 | 0.0075 |
| *atp*I | 6 | 741 | 0.81 | 0.0032 | *psb*I | 2 | 108 | 1.85 | 0.0062 |
| *ndh*A | 27 | 1089 | 2.48 | 0.0068 | *psb*J | 2 | 120 | 1.67 | 0.0056 |
| *ndh*B | 4 | 1530 | 0.26 | 0.0006 | *psb*K | 6 | 183 | 3.28 | 0.0097 |
| *ndh*C | 5 | 360 | 1.39 | 0.0043 | *psb*L | 1 | 114 | 0.88 | 0.0045 |
| *ndh*E | 1 | 303 | 0.33 | 0.0005 | *psb*M | 1 | 111 | 0.9 | 0.0015 |
| *ndh*G | 9 | 528 | 1.7 | 0.0047 | *psb*N | 0 | 129 | 0 | 0 |
| *ndh*H | 23 | 1179 | 1.95 | 0.0064 | *psb*T | 2 | 105 | 1.9 | 0.0039 |
| *ndh*I | 8 | 498 | 1.61 | 0.0046 | *psb*Z | 2 | 186 | 1.08 | 0.0017 |
| *ndh*J | 11 | 492 | 2.24 | 0.0080 | *rpl*14 | 8 | 366 | 2.19 | 0.0078 |
| *ndh*K | 17 | 849 | 2 | 0.0057 | *rpl*16 | 4 | 405 | 0.99 | 0.0018 |
| *acc*D | 41 | 1476 | 2.78 | 0.0087 | *rpl*2 | 3 | 825 | 0.36 | 0.0006 |
| *ccs*A | 34 | 972 | 3.5 | 0.0112 | *rpl*20 | 5 | 384 | 1.3 | 0.0048 |
| *cem*A | 14 | 720 | 1.94 | 0.0041 | *rpl*22 | 19 | 453 | 4.19 | 0.0138 |
| *clp*P | 8 | 588 | 1.36 | 0.0042 | *rpl*23 | 0 | 279 | 0 | 0 |
| *mat*K | 56 | 1542 | 3.63 | 0.0104 | *rpl32* | 10 | 174 | 5.75 | 0.0176 |
| infA | 2 | 231 | 0.87 | 0.0029 | *rpl*33 | 2 | 198 | 1.01 | 0.0035 |
| *ycf*1 | 327 | 5682 | 5.76 | 0.0190 | *rpl*36 | 2 | 111 | 1.8 | 0.0060 |
| *ycf*15 | 0 | 246 | 0 | 0 | *rpo*A | 34 | 1011 | 3.36 | 0.010 |
| *ycf*2 | 29 | 6819 | 0.43 | 0.0013 | *rpo*B | 48 | 3210 | 1.5 | 0.0041 |
| *ycf*3 | 3 | 507 | 0.59 | 0.0021 | *rpo*C1 | 28 | 2073 | 1.35 | 0.0036 |
| *ycf*4 | 15 | 552 | 2.72 | 0.0095 | *rpo*C2 | 77 | 4203 | 1.83 | 0.0049 |
| *pet*A | 19 | 960 | 1.98 | 0.0048 | *rps*11 | 5 | 414 | 1.21 | 0.0025 |
| *pet*B | 5 | 648 | 0.77 | 0.0024 | *rps*12 | 11 | 378 | 2.91 | 0.0080 |
| *pet*D | 9 | 486 | 1.85 | 0.0055 | *rps*14 | 3 | 300 | 1 | 0.0027 |
| *pet*G | 1 | 111 | 0.9 | 0.0014 | *rps*15 | 6 | 270 | 2.22 | 0.0061 |
| *pet*L | 2 | 93 | 2.15 | 0.0072 | *rps*16 | 13 | 264 | 4.92 | 0.0168 |
| *pet*N | 0 | 87 | 0 | 0 | *rps*18 | 5 | 303 | 1.65 | 0.0033 |
| *psa*A | 23 | 2250 | 1.02 | 0.0028 | *rps*19 | 7 | 276 | 2.54 | 0.0062 |
| *psa*B | 20 | 2202 | 0.91 | 0.0028 | *rps*2 | 13 | 714 | 1.82 | 0.0051 |
| *psa*C | 3 | 243 | 1.23 | 0.0049 | *rps*3 | 18 | 660 | 2.73 | 0.0075 |
| *psa*I | 2 | 108 | 1.85 | 0.0040 | *rps*4 | 9 | 603 | 1.49 | 0.0062 |
| *psa*J | 4 | 132 | 3.03 | 0.0066 | *rps*7 | 1 | 465 | 0.22 | 0.0012 |
| *psb*A | 13 | 1056 | 1.23 | 0.0040 | *rps*8 | 10 | 408 | 2.45 | 0.0076 |
| *psb*B | 14 | 1524 | 0.92 | 0.0028 | *rbc*L | 28 | 1461 | 1.92 | 0.0065 |

Note. PV: Percent variability; Pi: Nucleotide diversity.

**Table S3** dN, dS and dN/dS values of 78 orthologous genes identified in 14 *Scutellaria* Plants.

| Genes | dN | dS | dN/dS | Genes | dN | dS | dN/dS |
| --- | --- | --- | --- | --- | --- | --- | --- |
| *atp*A | 0.0042 | 0.0453 | 0.0927 | *psb*C | 0.0009 | 0.0307 | 0.0293 |
| *atp*B | 0.0056 | 0.0530 | 0.1057 | *psb*D | 0.0013 | 0.0385 | 0.0338 |
| *atp*E | 0.0064 | 0.0641 | 0.0998 | *psb*E | 0 | 0.0001 | 0 |
| *atp*F | 0.0073 | 0.0157 | 0.4650 | *psb*F | 0.0124 | 0.0314 | 0.3949 |
| *atp*H | 0.0056 | 0.0546 | 0.1026 | *psb*H | 0.0062 | 0.1023 | 0.0606 |
| *atp*I | 0 | 0.0363 | 0 | *psb*I | 0.0126 | 0.0375 | 0.3360 |
| *ndh*A | 0.0203 | 0.0828 | 0.2452 | *psb*J | 0.0109 | 0.0419 | 0.2601 |
| *ndh*B | 0.0021 | 0.0048 | 0.4375 | *psb*K | 0.0432 | 0 | / |
| *ndh*C | 0.0074 | 0.0508 | 0.1457 | *psb*L | 0 | 0.1035 | 0 |
| *ndh*E | 0.0040 | 0 | / | *psb*M | 0.0129 | 0 | / |
| *ndh*G | 0.0119 | 0.0408 | 0.2917 | *psb*N | 0 | 0.0001 | 0 |
| *ndh*H | 0.0079 | 0.0640 | 0.1234 | *psb*T | 0 | 0.0578 | 0 |
| *ndh*I | 0.0105 | 0.0474 | 0.2215 | *psb*Z | 0.0149 | 0 | / |
| *ndh*J | 0.0170 | 0.0492 | 0.3455 | *rpl*14 | 0.0178 | 0.0648 | 0.2747 |
| *ndh*K | 0.0210 | 0.0536 | 0.3918 | *rpl*16 | 0.0031 | 0.0508 | 0.0610 |
| *acc*D | 0.0254 | 0.0472 | 0.5381 | *rpl*2 | 0 | 0.0197 | 0 |
| *ccs*A | 0.0355 | 0.0530 | 0.6698 | *rpl*20 | 0.0070 | 0.0330 | 0.2121 |
| *cem*A | 0.0133 | 0.0149 | 0.8926 | *rpl*22 | 0.0591 | 0.1105 | 0.5348 |
| *clp*P | 0.0046 | 0.0436 | 0.1055 | *rpl*23 | 0 | 0.0001 | 0 |
| *mat*K | 0.0360 | 0.0562 | 0.6406 | *rpl*32 | 0.0088 | 0.2896 | 0.0304 |
| infA | 0 | 0.0313 | 0 | *rpl*33 | 0.0082 | 0.0138 | 0.5942 |
| *ycf*1 | 0.0604 | 0.0897 | 0.6734 | *rpl*36 | 0 | 0.0886 | 0 |
| *ycf*15 | 0 | 0.0001 | 0 | *rpo*A | 0.0280 | 0.0755 | 0.3709 |
| *ycf*2 | 0.0037 | 0.0093 | 0.3978 | *rpo*B | 0.0076 | 0.0489 | 0.1554 |
| *ycf*3 | 0.0095 | 0.0134 | 0.7090 | *rpo*C1 | 0.0070 | 0.0371 | 0.1887 |
| *ycf*4 | 0.0096 | 0.0891 | 0.1077 | *rpo*C2 | 0.0147 | 0.0394 | 0.3731 |
| *pet*A | 0.0111 | 0.05 | 0.2220 | *rps*11 | 0 | 0.0634 | 0 |
| *pet*B | 0 | 0.0372 | 0 | *rps*12 | 0.0652 | 0.0366 | 1.7814 |
| *pet*D | 0 | 0.0820 | 0 | *rps*14 | 0.0083 | 0.0179 | 0.4637 |
| *pet*G | 0 | 0.0553 | 0 | *rps*15 | 0.0142 | 0.0551 | 0.2577 |
| *pet*L | 0.0305 | 0 | / | *rps*16 | 0.0284 | 0.1937 | 0.1466 |
| *pet*N | 0 | 0.0001 | 0 | *rps*18 | 0.0126 | 0.0345 | 0.3652 |
| *psa*A | 0.0025 | 0.0310 | 0.0806 | *rps*19 | 0.0138 | 0.0841 | 0.1641 |
| *psa*B | 0.0017 | 0.0315 | 0.0540 | *rps*2 | 0.0091 | 0.0542 | 0.1679 |
| *psa*C | 0 | 0.0464 | 0 | *rps*3 | 0.0193 | 0.0620 | 0.3113 |
| *psa*I | 0 | 0.0755 | 0 | *rps*4 | 0.0109 | 0.0290 | 0.3759 |
| *psa*J | 0.0109 | 0.1006 | 0.1083 | *rps*7 | 0.0027 | 0 | / |
| *psb*A | 0.0012 | 0.0561 | 0.0214 | *rps*8 | 0.01 | 0.0733 | 0.1364 |
| *psb*B | 0.0017 | 0.0373 | 0.0456 | *rbc*L | 0.0171 | 0.0396 | 0.4318 |

Note. dN: non-synonymous substitution rate; dS: synonymous substitution rate.
